# First Report on Comparative Essential Oil Profile of Stem and Leaves of *Blepharispermum hirtum* Oliver and Their Antidiabetic and Anticancer Effects

**DOI:** 10.3390/metabo12100907

**Published:** 2022-09-26

**Authors:** Muddaser Shah, Saif Khalfan Al-Housni, Faizullah Khan, Saeed Ullah, Jamal Nasser Al-Sabahi, Ajmal Khan, Balqees Essa Mohammed Al-Yahyaei, Houda Al-Ruqaishi, Najeeb Ur Rehman, Ahmed Al-Harrasi

**Affiliations:** 1 Natural & Medical Sciences Research Center, University of Nizwa, Nizwa 616, Oman; 2 Department of Botany, Abdul Wali Khan University Mardan, Mardan 23200, Pakistan; 3 Department of Pharmacy, Abdul Wali Khan University Mardan, Mardan 23200, Pakistan; 4 Hussian Ebrahim Jamal Research Institute of Chemistry, International Center for Chemical and Biological Sciences, University of Karachi, Karachi 75270, Pakistan; 5 Central Instrument Laboratory, College of Agriculture and Marine Sciences, Sultan Qaboos University, Muscat 123, Oman

**Keywords:** *Blepharispermum hirtum*, Asteraceae, GS-MS analysis, essential oils, triple-negative breast cancer, α-glucosidase

## Abstract

The current research was designed to explore the *Blepharispermum hirtum* Oliver (Asteraceae) stem and leaves essential oil (EO) composition extracted through hydro-distillation using gas chromatography-mass spectrometry (GC-MS) analysis for the first time. The EOs of the stem and leaves of *B. hirtum* were comparatively studied for the in vitro antidiabetic and anticancer potential using in vitro α-glucosidase and an MTT inhibition assay, respectively. In both of the tested samples, the same number of fifty-eight compounds were identified and contributed 93.88% and 89.07% of the total oil composition in the EOs of the stem and leaves of *B. hirtum* correspondingly. However, camphene was observed as a major compound (23.63%) in the stem EO, followed by β-selinene (5.33%) and β-elemene (4.66%) and laevo-β-pinene (4.38%). While in the EO of the leaves, the dominant compound was found to be 24-norursa-3,12-diene (9.08%), followed by β-eudesmol (7.81%), β-selinene (7.26%), thunbergol (5.84%), and caryophyllene oxide (5.62%). Significant antidiabetic potential was observed with an IC_50_ of 2.10 ± 0.57 µg/mL by the stem compared to the EO of the leaves of *B. hirtum*, having an IC_50_ of 4.30 ± 1.56 µg/mL when equated with acarbose (IC_50_ = 377.71 ± 1.34 µg/mL). Furthermore, the EOs offered considerable cytotoxic capabilities for MDA-MB-231. However, the EO of the leaves presented an IC_50_ = 88.4 ± 0.5 μg/mL compared to the EO of the stem of *B. hirtum* against the triple-negative breast cancer (MDA-MB-231) cell lines with an IC_50_ = 123.6 ± 0.8 μg/mL. However, the EOs were also treated with the human breast epithelial (MCF-10A) cell line, and from the results, it has been concluded that these oils did not produce much harm to the normal cell lines. Hence, the present research proved that the EOs of *B. hirtum* might be used to cure diabetes mellitus and human breast cancer. Moreover, further studies are considered to be necessary to isolate the responsible bioactive constituents to devise drugs for the observed activities.

## 1. Introduction

Medicinal plants and their products serve as both traditional and commercial alternative innovative remedies [1]. Due to the efficacy and lower adverse effects, the demand for herbal therapies has increased. Plant based natural products, comprising essential oils (EOs) have gained attention due to their usage in foodstuff, cosmetics, and pharmaceutical productions. Constituting a range of several lipophilic and extremely volatile constituents, obtained from an extensive range of diverse chemical classes, EOs are attributed to multiple health benefits: analgesic, anti-inflammatory, antioxidant, antimicrobial, anticancer, and antidiabetic [2]. 

Diabetes mellitus is considered as a world health issue, linked to two key features including insufficient insulin secretion or insensitivity to their action. Their high rate of prevalence reflects its severity, and according to the projected statistics of WHO, more than 422 million people have diabetes, 1.5 million deaths are directly attributed to diabetes each year, and the prevalence ratio will increase to 693 million by 2045 [1,3]. The diabetes might lead to several other complications such as polyuria, polydipsia, impaired vision, and skin infections [4]. Therefore, strategies need to be developed to combat these drawbacks. In this context, α-glucosidase (EC 3.2.1.20) has become a promising target for the treatment of diabetes mellitus. The inhibition of these carbohydrates’ key metabolic enzymes slows down carbohydrate digestion, resulting in the low absorption of glucose, leading to normalizing the blood glucose levels. Hence, the investigation of new anti-diabetic agents using natural sources is currently of need because of their non-cytotoxic effects [5,6,7]. 

Increased interest of users concerning pharmacologically effective plant-based natural products (NPs) as substitute therapies to treat cancer has increased the attention of scientists worldwide [4]. However, an intensifying significance has been observed recently that EOs act as an anticancer medication to overcome the development of multidrug resistance and critical harmful effects linked with available antitumor remedies [5]. Therefore, due to the key role of EOs in cytotoxic therapy, the EOs of unreported plants might be used as a complementary remedy [6]. 

*Blepharispermum hirtum* Oliver (family: Asteraceae) is a naturally growing tree, about 2 m in height, and is endemic to Dhofar (Oman). The plant has very broad and soft leaves with a basic inflorescence containing a capitulum of white flowers. The genus *Blepharispermum* comprises 15 species, all of which are shrubs, except for *B hirtum. Blepharispermum* species are distributed over different regions of Africa, the Arabian Peninsula, and India [7]. Decoctions as well as the root powder of *B. subsessile* have been used by local practitioners in India for the treatment of various health ailments used in nervous disorders, while the whole plant is used in diarrhea, stomach ache, rheumatic affections, skin diseases, eye troubles, anti-inflammatory diseases, and irregular menstruation [8,9,10]. Recently, Fatope et al., [9] reported ent-kaurene diterpenoids with larvicidal and antimicrobial activity. It also has promising potential to resist microbes and antifeedant significance [9]. The genus *Blepharispermum* is an affluent basis for many bioactive ingredients including dimethyl isoencecalin and 5-hydroxy-6-acetyl-2-hydroxymethyl-2-methyl chromene [10]. 

Furthermore, the reported literature of EOs and the traditional uses of the plants growing in Oman have been noticed to have promising potential to cure diabetes and cancer [11,12]. Natural products derived from plants and plant products that have been traditionally used to treat various diseases including cancer and diabetes have advantages in drug discovery [13]. Thus, the current study was designed to profile the constituents of the EOs and determine the in vitro antidiabetic and cytotoxic significance of *B. hirtum*. Hence, the recent study will update the literature on the genus *Blepharispermum* and report on the EOs of *B. hirtum* for the first time.

## 2. Materials and Methods 

### 2.1. General Instrumentation

The MDA-MB-231 and MCF-7 cell lines were acquired from the American Type Culture Collection (ATCC) and MCF-10A was purchased from the Iranian Biological Resource Center (IBRC) (Tehran, Iran). GC-MS was conducted on a gas chromatography-mass spectrometer (GC-MS-QP2010, Shimadzu Kyoto, Japan). The α-glucosidase enzyme (EC 3.2.1.20, Sigma-Aldrich, Darmstadt, Germany) and spectrophotometer (xMark™ Microplate Spectrophotometer, Bio-Rad, Hercules, CA, USA) were used for the α-glucosidase activity. A High-Speed Multifunctional Grander (Grand Household, Code. GR-SCG350H) was used for the grinding of the plant. Analytical grade reagents were used in the current study. 

### 2.2. Collection and Identification of Plant Materials

The whole plant material of *B. hirtum* (8.7 kg) was collected from Salalah, the Dhofar region of Oman (April–May 2020). After identification by the plant taxonomist (Syed Abdullah Gilani, Department of Biological Sciences and Chemistry, University of Nizwa, Nizwa, Oman), the leaves (4.0 kg) were separated from the stems (4.2 kg) and placed under shade at room temperature for dryness. The dried samples were ground into fine powder (50–300 mesh) using a stainless-steel blender. A voucher specimen of *B. hirtum* (BHO-03/2020) was deposited in the herbarium of the Natural and Medical Sciences Research Center, University of Nizwa, Oman. 

### 2.3. Essential Oils Extraction

The essential oils extracted through hydro-distillation from the leaves and stem of the *B. hirtum* yielded 1.2 g (0.052%) and 0.95 g (0.045%), respectively, using a Clevenger-type apparatus (three times for at least 6 h) and were observed to be yellow-colored [14,15]. A known quantity of the EOs was collected, dried over anhydrous sodium sulfate (Na_2_SO_4_), and kept in the refrigerator at 4 °C until further GC-MS analysis and in vitro antidiabetic and cytotoxic assays. 

### 2.4. GC-MS Analysis 

The chemical constituents in the stem and leaf samples of the understudy plant were determined through the Perkin Elmer Clarus (PEC) 600 GC System (Perkin Elmer, Waltham, MA, USA) using gas chromatography-mass spectrometry (GC/MS) analysis. The GC/MS instrument was coupled with an Rtx-5MS capillary column (30 m × 0.25 mm, 0.25 μm film thickness) at 260 °C, connected to a PEC 600 mass spectrometer (MS). Electron multiplier (EM) voltage was achieved from autotune with 70 eV ionizing energy (IE). The carrier gas was helium (99.9999%) with a flow rate of 1 mL/min, while temperatures of 260 °C and 280 °C were used for the injection, transfer line, and ion source, respectively, during the whole analysis. The oven temperature was kept at 60 °C, holds for 1 min, at a flow rate of 4 °C/min–260 °C, and stood for 4 min. The essential oil solution (1 μL) was injected with a split ratio of 10:1. The complete chromatographic data were obtained by accumulating the full-scan mass spectra in the range of 45–550 amu. Furthermore, the total processing time of the GC/MS analysis was 55 min.

#### Identification of the Components

The essential oils extracted from the leaves and stems of *B. hirtum* were identified by their respective chromatogram peaks obtained for each compound through GC-MS analysis. Some of the compounds were identified by comparing their mass spectra with the MS library database (NIST 2011 v.2.3). Compound identification was also made possible by comparing their retention times (Rt) with those of the pure authentic samples and by means of their retention index (RI), relative to the series of n-hydrocarbons [14,15,16]. 

### 2.5. In Vitro α-Glucosidase Inhibitory Assay

Evaluation of the α-glucosidase inhibitory significance of the essential oils of the tested samples proceeded at 37 °C using 0.5 mM phosphate buffer (pH 6.8) [9,10]. High to low doses of the tested samples including (60, 30, 15, 7.8, 3.90, and 1.95 µg/mL), respectively, were incubated with the enzyme (2 U/2 mL) in phosphate buffer for 15 min at 37 °C. After adding the 25 µL substrate, p-nitrophenyl-a-D-glucopyranoside (0.7 mM, final), a spectrophotometer was used to track the changes in absorbance at 400 nm for 30 min. DMSO-d6 (7.5 percent final) was used as a positive control. As a reference standard, acarbose (IC50 = 377.7 1.34 µg/mL) was employed. Furthermore, the IC_50_ was calculated by using EZ-fit software, as explained in the statistical analysis section by Equations (2) and (3).

### 2.6. In Vitro Cytotoxic Potential

In vitro cytotoxicity capacity of EOs was determined by performing an MTT (yellow tetrazolium salt, 3-(4,5-dimethylthizol-2-yl)-2,5-diphenyl tetrazolium bromide) assay by using an aggressive breast cancer cell line (MDA-MB-231) [17]. Human breast normal cell line MCF-10A was kept as a control in the study. Cells were cultured in Dulbecco’s modified Eagle medium (DMEM) supplemented with 10% FBS and 1% antibiotics (100 U/mL penicillin). The cells were seeded in a 96-well plate at a density of 1.0 × 10^4^ cells/well and incubated for 24 h at 37 °C in 5% CO_2_. The medium was discarded, and both cell lines were treated with different concentrations (3, 10, 30, 100, and 300 μg/mL) of plant EOs [18] after 48 h of incubation (Maher et al. [19]). A total of 20 μL of MTT solution (5 mg/mL) was pipetted into each well and incubated for another 4 h. The medium was later discarded, and the formazan precipitate was dissolved in DMSO. The absorbance of the mixtures was determined using a microplate reader at 570 nm. All experiments were performed in triplicate and the cytotoxicity was expressed as a percentage of cell viability compared to the untreated control cells [18]

(1) $$\begin{array}{c} \%\;\mathrm{Viability}=\frac{\mathrm{Absorbance}\;\mathrm{of}\;\mathrm{sample}}{\mathrm{Absorbance}\;\mathrm{of}\;\mathrm{control}}\times 100 \end{array}$$

### 2.7. Statistical Analysis

Excel and the SoftMax Pro package were used as the applications to examine the results for biological activity. The following formula was used to determine the % inhibition.

(2) $$\begin{array}{c} \%\;\mathrm{Inhibition}=100-\left(\frac{O.D_{test\;compound}}{O.D_{control}}\right)\times 100 \end{array}$$

All of the tested substances’ IC_50_ values were calculated using EZ-FIT (Perrella Scientific, Inc., Amherst, MA, USA). All experiments were carried out in triplicate to reduce the likelihood of mistakes, and differences in the results are reported as the standard error of mean values (SEM).

(3) $$\begin{array}{c} SE=\frac{\sigma }{\sqrt{n}} \end{array}$$

The cytotoxic activity was estimated via IBM SPSS Statistics 26 software and utilized to analyze the dose response and computation of IC_50_.

## 3. Results and Discussion

### 3.1. Composition of Essential Oil 

**Figure 1:**
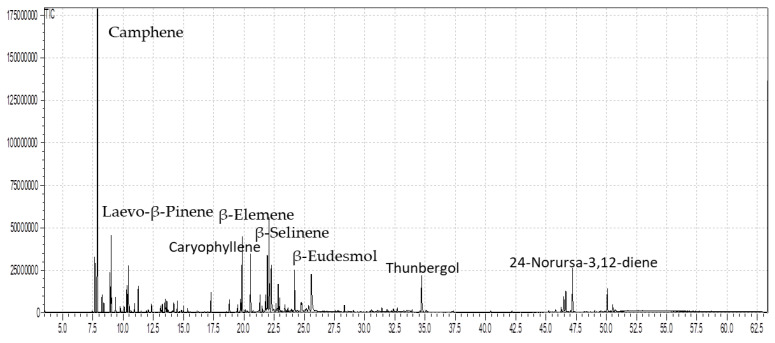
The GC chromatogram of the essential oils of the stem of *B. hirtum*.

**Figure 2:**
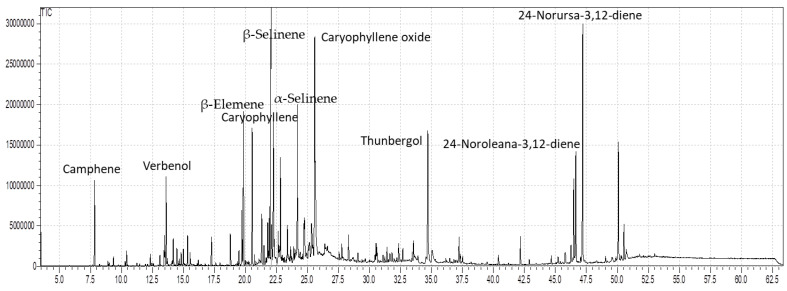
The GC chromatogram of the essential oils of the leaves of *B. hirtum*.

The role of essential oils in the therapy of human health complications from ancient times to date cannot be denied. The promising potential attributed to EOs is due to the presence of valuable ingredients. In the current study, through the GC-MS analysis, fifty-eight compounds were identified in the EOs of the stems and leaves of *B. hirtum* (Table 1). The compounds identified in the stem through GC-MS screening contributed 93.88% of the total oil composition among which camphene was noticed as the dominant compound having 23.63%, followed by β-selinene with 5.33%, β-elemene (4.66%), and laevo-β-pinene (4.38%) (Table 1 and Figure 1). While the same number of compounds were identified in the EOs of the leaves of the *B. hirtum* sample, which contributed 89.07% of the total composition, with major compounds of 24-norursa-3,12-diene at 9.08%, followed by β-eudesmol (7.81%), β-selinene (7.26%), thunbergol (5.84%), and caryophyllene oxide (5.62%) (Figure 1 and Figure 2). The compound camphene was earlier reported in the EOs of *Piper cernuum*, as presented by Girola et al. [20], while β-selinene was previously noticed in *Litsea cubeba* and *Lanthana camara*, as stated by Si et al. [21] and Sarma et al. [22], respectively. Furthermore, our data consented to the outcomes elaborated by Quassinti et al. [23] in *Hypericum hircinum* and *Ferulago macrocarpa*, as described by Sajjadi et al. [24]. In addition, our data are also supported by the outcomes earlier reported by Akpulat et al. [25] and Hulley et al. [26] in the EOs of some plants belonging to the family Asteraceae, which might be due to the presence of the common chemical ingredients. However, our findings do not match the EOs reported by Mejia et al. [27] in *Brassica nigra* and also with the literature documented by Oroojalian et al. [28] in some Apiaceae species. Many factors are responsible for the variation among the contents present within a plant species including the difference in plant family and environmental gradients [29]. 

### 3.2. In Vitro Antidiabetic Significance

**Figure 3:**
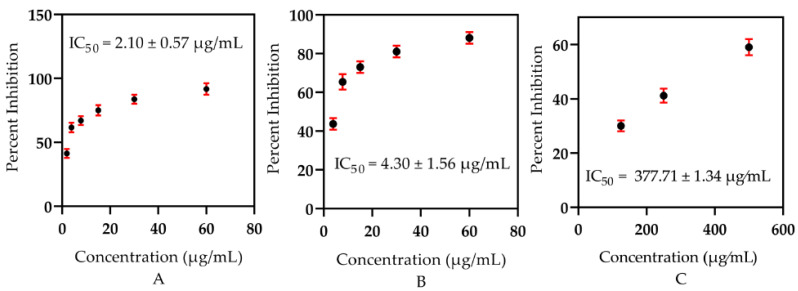
The in vitro antidiabetic significance of *B. hirtum* essential oils: (**A**) Stem, (**B**) leaves, and (**C**) standard acarbose.

It is very clear that in recent times, natural products have been considered as untapped diamonds because of their invaluable medicinal use and lower side effects. The recent studies were designed to keep these key features of new drug candidates. The reported pharmacotherapeutic importance of EOs in the treatment of diabetes encouraged us to identify anti-diabetic agents via evaluating natural resources [30]. Therefore, in the current studies, two samples of EOs extracted from *B. hirtum* were subjected to, due to their crucial role in the inhibition of the key anti-diabetic targeted enzyme, α-glucosidase. Interestingly, both samples displayed overwhelming anti-diabetic potential with very high potency IC_50_ = 2.10 ± 0.57 µg/mL (stem) and 4.30 ± 1.56 µg/mL (leaves) when compared with the marketed drug acarbose IC_50_ = 377.71 ± 1.34 µg/mL (Figure 3). This invaluable high potency of these natural products further showed and strengthened their role as anti-diabetic agents. The stem EOs contained camphene in a higher quantity (23.63%) compared to the leaves (2.19%), which has a significant role in curing diabetes, as reflected in the literature stated by Mishra et al. [31] and Hachlafi et al. [32], and this might be the reason for which the EOs of the stem depicted a significant capacity to act as an antidiabetic agent. In addition, our findings were in agreement with the data reported by Majouli et al. [33] for *Hertia cheirifolia* and Ceylan et al. [34], which documented the significance of *Thymus spathulifolius* due to the presence of common constituents and the same technique used in the mentioned plant species and understudy plant samples. However, our results did not match the previously described outcome of Ahmad [35] for *M. spicata* and Basak et al. [36], which revealed the significance of the EOs of *Laurus nobilis.* Variation in the capacity of the plants mainly depends upon the chemical ingredients that might be altered due to the edaphic, climatic, quality, and availability of water, as stated by Shah et al. [37], and is also affected by the elemental and other ingredients present in the water available for plants.

### 3.3. In Vitro Cytotoxicity Capacity

The cytotoxic potential of the tested samples of *B. hirtum* EOs was evaluated from low to high doses using human breast cancer cell line MDA-MB-231 compared to human normal breast epithelial cell lines; Michigan cancer foundation (MCF) MCF-10A was used as a control in the experiment. The MTT [3-(4,5-dimethylthiazol-2-yl)-2,5-diphenyltetrazolium bromide] assay was used to determine the decrease in the cancer cell viability induced by cytotoxic agents. For MDA-MB-231, the IC_50_ values, % inhibition, and viability of the tested EOs are presented in Table 2. Our findings show that the EOs of the leaves and stem have promising capabilities against MDA-MB-231 cells with IC_50_ values of 88.4 ± 0.5 and 123.6 ± 0.8 μg/mL, respectively. To determine whether the cytotoxic effects of the oils were selective for malignant cells in comparison to the non-malignant cells, the non-tumorigenic MCF-10A cells were screened through the tested EOs from low to high doses (3, 10, 30, 100, and 300 μg/mL) in a similar manner as the cancer cells. After the MTT assay, the results of the % inhibition and viability for the MCF-10A cell lines by essential oils are presented in Table 3. The results showed that these cells were less susceptible to the actions of the essential oil, particularly at a higher dose of 300 μg/mL. The data in this study revealed that the triple negative MDA-MB-231 cells, which bear an aggressive phenotype, responded more favorably to EOs, and showed greater cytotoxicity. The significant potential for cytotoxicity was observed when non-tumorigenic MCF-10A cells were exposed to this plant’s EOs, which suggested that EOs have the potential in offering promising treatment for patients with breast cancer. Some valuable constituents were noticed in the understudy plant samples due to which they offered promising potential cancer therapy. Our findings agreed with the report described by Ortiz et al. [38] of the plant species belonging to the genus *Santalum* and Furtoda et al. [39] in the *Blepharocalyx salicifolius.* Our findings also favor the study of Loizzo et al. [40], who described the significance of the EOs of some plants of the family Lamiaceae and Lauraceae.

## 4. Conclusions

The comparative analysis of the *B. hirtum* stem and leaves EOs revealed that the understudy plant is an affluent source of responsible bioactive chemical constituents that are intended to produce as useful properties as the plant. Fifty-eight constituents were observed in the EOs of the stem and leaves of *B. hirtum* and contributed 93.88% and 89.07% of the total amount, respectively. Camphene was observed as a major compound (23.63%), followed by β-selinene (5.33%), β-elemene (4.66%), and laevo-β-pinene (4.38%) in the stem EO. While the 24-norursa-3,12-diene (9.08%), β-eudesmol (7.81%), β-selinene (7.26%), thunbergol (5.84%), and caryophyllene oxide (5.62%) were noted as the dominant constituents. Considerable potential to cure diabetes was offered by the tested samples compared to the standard. Moreover, the EOs of *B. hirtum* produced significant cytotoxicity effects against the breast cancer cell line MDA-MB-231 and were non-toxic to the normal cell line MCF-10A. The occurrence of the chemical constituents and promising α-glucosidase and cytotoxic activities of the EOs validate their pharmaceutical and nutraceutical importance. Hence, the analysis revealed that *B. hirtum* EOs can be used as an alternative promising natural remedy to cure diabetes mellitus and cancer.

## Figures and Tables

**Table 1:** The GC-MS analysis of the EOs of *Blepharispermum hirtum* Oliver.

S. No.	Compounds	RT_min_	RI_cal_	RI_rep_	% Stem	% Leaves
1	5,5-Dimethyl-1-vinylbicyclo [2.1.1] hexane	7.44	927	920	0.12	0.03
2	3-Thujene	7.65	935	928	3.11	0.06
3	Camphene	7.88	944	935	23.63	2.19
4	2,4(10)-Thujadiene	8.39	963	957	0.53	0.03
5	Sabinene	8.91	982	964	2.21	0.14
6	Laevo-β-Pinene	9.01	986	978	4.38	0.12
7	β-Myrcene	9.37	999	981	0.91	0.25
8	α-Phellandrene	9.75	1013	997	0.39	0.05
9	3-Carene	9.92	1019	1005	0.11	0.04
10	p-Cymene	10.30	1033	1011	1.46	0.14
11	D-Limonene	10.42	1037	1018	2.79	0.46
12	γ-Terpinene	11.25	1067	1047	1.51	0.11
13	Linalool	12.34	1106	1082	0.49	0.32
14	Perillen	12.40	1108	1086	0.04	0.08
15	α-Campholenal	13.10	1134	1102	0.42	0.31
16	2,9-Dimethyl-5-decyne	13.10	1136	1103	0.31	0.03
17	L-Pinocarveol	13.46	1147	1108	0.85	0.84
18	cis-Verbenol	13.52	1149	1110	0.25	0.36
19	trans-Verbenol	13.61	1153	1128	0.71	2.51
20	p-Mentha-1,5-dien-8-ol	14.18	1174	1148	0.56	0.69
21	Terpinen-4-ol	14.47	1185	1175	0.67	0.46
22	Myrtenol	15.00	1205	1174	0.45	0.49
23	Levoverbenone	15.34	1218	1191	0.26	0.86
24	cis-Carveol	15.54	1226	1208	0.08	0.37
25	Bornyl acetate	17.26	1292	1269	1.23	0.83
26	α-Terpinyl acetate	18.78	1354	1322	0.91	0.97
27	Copaene	19.48	1383	1376	0.47	0.44
28	β-Bourbonene	19.71	1392	1386	0.84	1.57
29	β-Elemene	19.84	1398	1398	4.66	4.52
30	Caryophyllene	20.54	1428	1421	3.73	4.35
31	Humulene	21.32	1462	1454	1.31	1.55
32	Alloaromadendrene	21.49	1469	1459	0.39	0.59
33	γ-Muurolene	21.80	1483	1471	1.05	1.01
34	Germacrene D	21.94	1489	1480	3.26	1.31
35	β-Selinene	22.08	1495	1509	5.33	7.26
36	α-Selinene	22.26	1503	1500	2.92	4.63
37	Cubebol	22.64	1521	1512	0.33	0.99
38	δ-Cadinene	22.82	1529	1514	1.59	2.93
39	Elemol	23.38	1554	1535	0.63	1.42
40	Germacrene D-4-ol	23.99	1582	1570	0.09	0.31
41	Caryophyllene oxide	24.19	1991	1575	2.89	5.62
42	Humulene 1,2-epoxide	24.743	1617	1596	0.61	1.16
43	γ-Eudesmol	24.786	1619	1627	0.58	1.18
44	Cubenol	25.09	1634	1631	0.13	0.38
45	tau-Cadinol	25.341	1946	1637	0.77	1.78
46	β-Eudesmol	25.57	1657	1644	2.73	7.81
47	Benzyl Benzoate	27.77	1767	1765	0.12	0.54
48	α-Phellandrene, dimer	28.32	1794	1801	0.43	0.76
49	m-Camphorene	31.11	1945	1960	0.09	0.31
50	Cembrene A	31.41	1961	1970	0.32	0.58
51	p-Camphorene	31.68	1978	1977	0.08	0.41
52	Geranyl-α-terpinene	32.21	2007	1990	0.05	0.13
53	Verticillol	32.70	2036	2036	0.29	0.38
54	Cembrenol	34.55	2046	2161	0.25	0.29
55	Thunbergol	34.71	2156	2173	3.32	5.84
56	24-Norursa-3,9(11),12-triene	46.48	2156	3042	1.23	3.17
57	24-Noroleana-3,12-diene	46.645	3013	3057	1.55	4.03
58	24-Norursa-3,12-diene	47.198	3060	3105	3.46	9.08
	Total % of the identified compounds				93.88	89.07

RI_calc_ = Retention index calculated. RI_rep_ = Retention index obtained from database (NIST, 2011). RT = Retention time (min).

**Table 2:** The % viability and inhibition of *B. hirtum* essential oil on the breast cancer cell line MDA-MB-231.

Tested Samples	Conc. (μg/mL)	% Viability	% Inhibition	IC50 (μg/mL)
Leaves	3	94.33	5.66	88.4 ± 0.5
	10	82.41	17.58	
	30	71.09	28.90	
	100	47.85	52.14	
	300	26.26	73.73	
Stem	3	96.49	3.50	123.6 ± 0.8
	10	85.43	14.56	
	30	72.65	27.34	
	100	53.04	46.95	
	300	37.05	62.94	

**Table 3:** The % Viability and inhibition of *B. hirtum* essential oil on the normal breast cell line MCF-10A.

Tested Samples	Conc (μg/mL)	% Viability	% Inhibition	IC_50_ (μg/mL)
Leaves	3	96.29	3.70	>300
	10	93.04	6.99	
	30	89.34	10.65	
	100	86.96	13.03	
	300	78.07	23.99	
Stem	3	97.50	2.49	>300
	10	95.28	4.71	
	30	89.93	10.06	
	100	85.10	14.89	
	300	79.22	20.77	

## Data Availability

The data presented in this study are available in the article.
